# Supplementary material for: Succinylation of Polyallylamine: Influence on Biological Efficacy and the Formation of Electrospun Fibers
Source: Polymers (Basel). 2021 Aug 24;13(17):2840. doi: 10.3390/polym13172840 (PMC8433649; doi:10.3390/polym13172840)
Supplement: Supplementary file 1 [file polymers-13-02840-s001.zip › polymers-1332041-supplementary.pdf]

Supplementary information (ESI)

Succinylation of Polyallylamine: Influence on Biological Efficacy and the Formation of Electrospun Fibers

Lucija Jurko [1], Matej Bračič [1], Silvo Hribernik [1,2], Damjan Makuc [3,4], Janez Plavec [3,4,5], Filip Jerenec [6], Sonja Žabkar [7], Nenad Gubeljak [6], Alja Štern [7] and Rupert Kargl [1,2,8*]

XRD measurements

According to previous research on succinylation of polymers, in some cases, there was a difference in the crystallinity between the product and the base polymer [1,2]. With the increase of the DS of PAA, a difference in the macroscopic morphology of each product could be observed (Figure S1). This was especially seen when comparing the base polymer (PAA) to samples with a high DS (PAA-5SA and PAA-10SA).

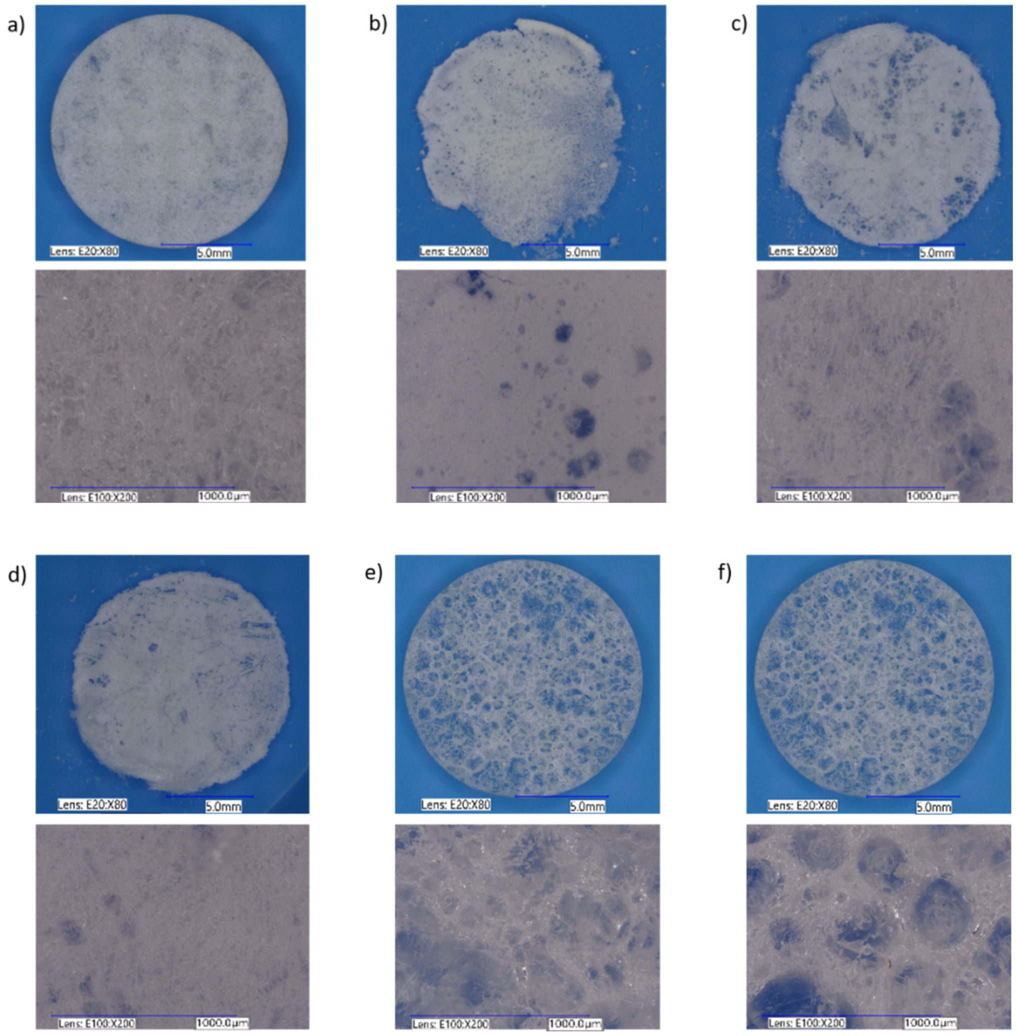

Figure S1: Images of polymer powders: *a) PAA, b) PAA-0.5SA, c) PAA -1SA, d) PAA-2.5SA, e) PAA-5SA , f) PAA-10SA, upper rows, photograph, lower rows light microscopy images.*

According to XRD measurements, crystallinity of the material did not change with increase DS and was calculated to be at approximately 22 % for each sample.

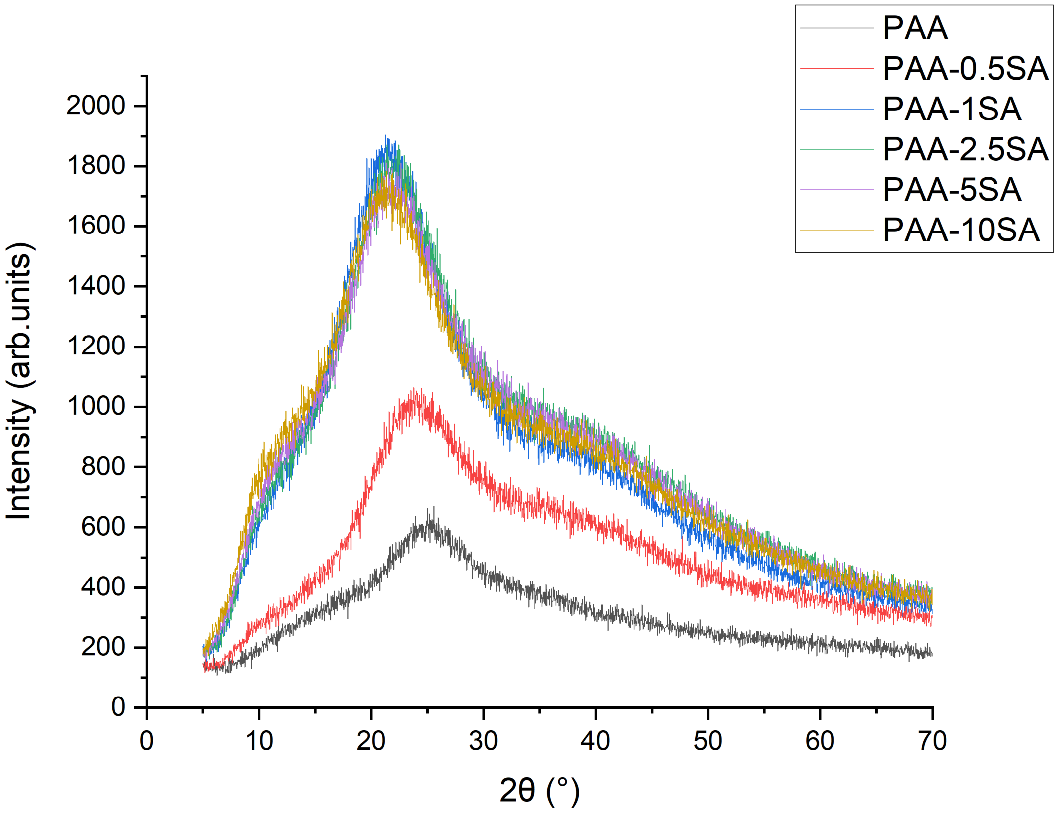

Figure S2: XRD spectra of polymers*: PAA, PAA-0.5SA, PAA -1SA, PAA-2.5SA, PAA-5SA, PAA-10SA*

Broad peaks of XRD spectra are in correspondence to the amorphous structure of polyallylamine hydrochloride with a 2 Θ peak between 23 and 28° in correspondence with literature [2,3,4].

Table S1 ***Antimicrobial properties of polymers against Gram positive S. aureus:*** *PAA and PAA-xSA samples. Symbols depict complete growth inhibition of the bacteria (-) highlighted in red, partial growth inhibition of the bacteria (+) highlighted in yellow, and non- inhibitory effect on the growth of the bacteria (++) highlighted in green.*

Staphylococcus aureus											
Concentration /mg mL^{-1}											
Sample	**20**	**10**	**5**	**2.5**	**1.25**	**0.62**	**0.31**	**0.16**	**0.08**	**0.04**	**0.02**
**PAA**	-	-	-	-	-	-	-	-	-	++	++
**PAA-1SA**	++	++	++	++	++	++	++	++	++	++	++
**PAA-2.5SA**	+	++	++	++	++	++	++	++	++	++	++
**PAA-5SA**	+	+	++	++	++	++	++	++	++	++	++
**PAA-10SA**	+	+	++	++	++	++	++	++	++	++	++

Table S2: Antimicrobial properties of polymers against P. aeruginosa: *Symbols depict complete growth inhibition of the bacteria (-) in red, partial growth inhibition of the bacteria (+) in yellow and non- inhibitory effect on the growth of the bacteria (++) in green.*

Pseudomonas aeruginosa											
Concentration /mg mL^{-1}											
Sample	**20**	**10**	**5**	**2.5**	**1.25**	**0.62**	**0.31**	**0.16**	**0.08**	**0.04**	**0.02**
**PAA**	-	-	-	-	-	-	-	-	-	+	++
**PAA-1SA**	++	++	++	++	++	++	++	++	++	++	++
**PAA-2.5SA**	++	++	++	++	++	++	++	++	++	++	++
**PAA-5SA**	++	++	++	++	++	++	++	++	++	++	++
**PAA-10SA**	++	++	++	++	++	++	++	++	++	++	++

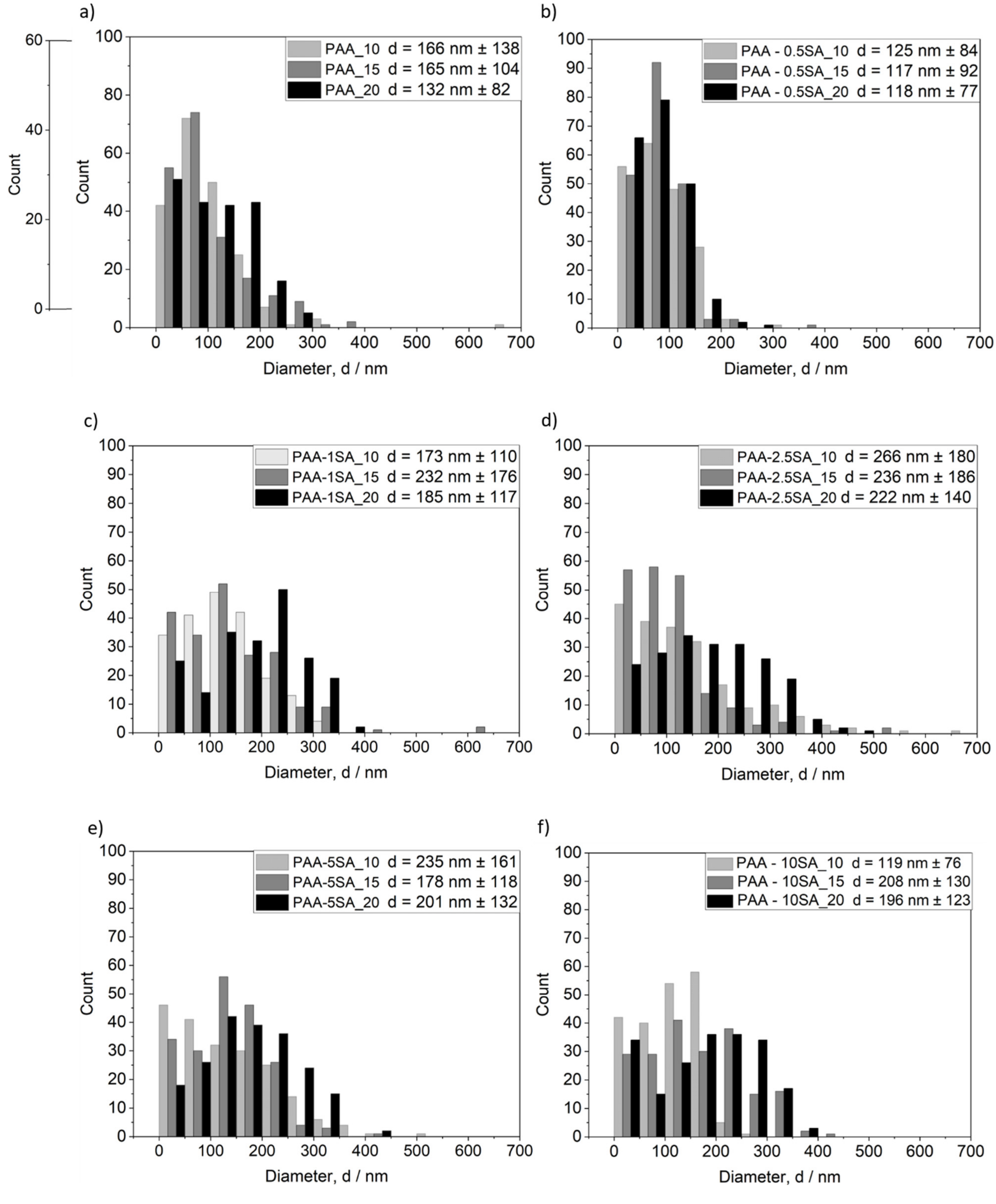

Figure S3: Fiber diameter histograms: *PVA fibers (above) with addition of a) PAA, b) PAA-0.5SA, c) PAA-1SA, d) PAA-2.5 SA, e) PAA-5SA and f) PAA-10SA. 200 measurements were made from three repetitions per polymer type.*

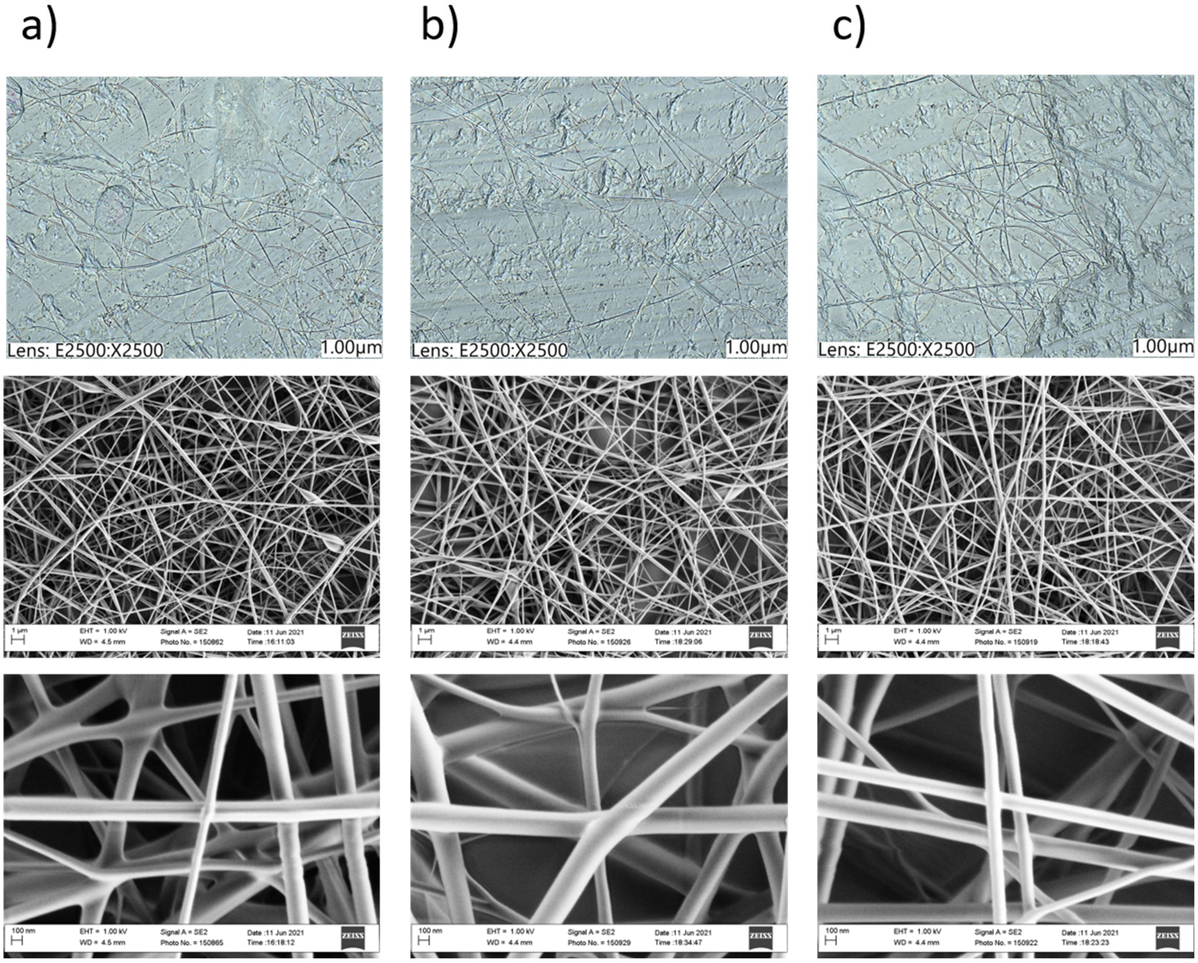
a)
b)
c)
Lens: E2500:X2500
1.00µm
Lens: E2500:X2500
1.00µm
Lens: E2500:X2500
1.00µm
1 µm EHT = 1.00 kV WD = 4.5 mm Signal A = SE2 Photo No. = 150862 Date :11 Jun 2021 Time :16:11:03 ZEISS
1 µm EHT = 1.00 kV WD = 4.4 mm Signal A = SE2 Photo No. = 150926 Date :11 Jun 2021 Time :18:29:06 ZEISS
1 µm EHT = 1.00 kV WD = 4.4 mm Signal A = SE2 Photo No. = 150919 Date :11 Jun 2021 Time :18:18:43 ZEISS
100 nm EHT = 1.00 kV WD = 4.5 mm Signal A = SE2 Photo No. = 150865 Date :11 Jun 2021 Time :16:18:12 ZEISS
100 nm EHT = 1.00 kV WD = 4.4 mm Signal A = SE2 Photo No. = 150929 Date :11 Jun 2021 Time :18:34:47 ZEISS
100 nm EHT = 1.00 kV WD = 4.4 mm Signal A = SE2 Photo No. = 150922 Date :11 Jun 2021 Time :18:23:23 ZEISS

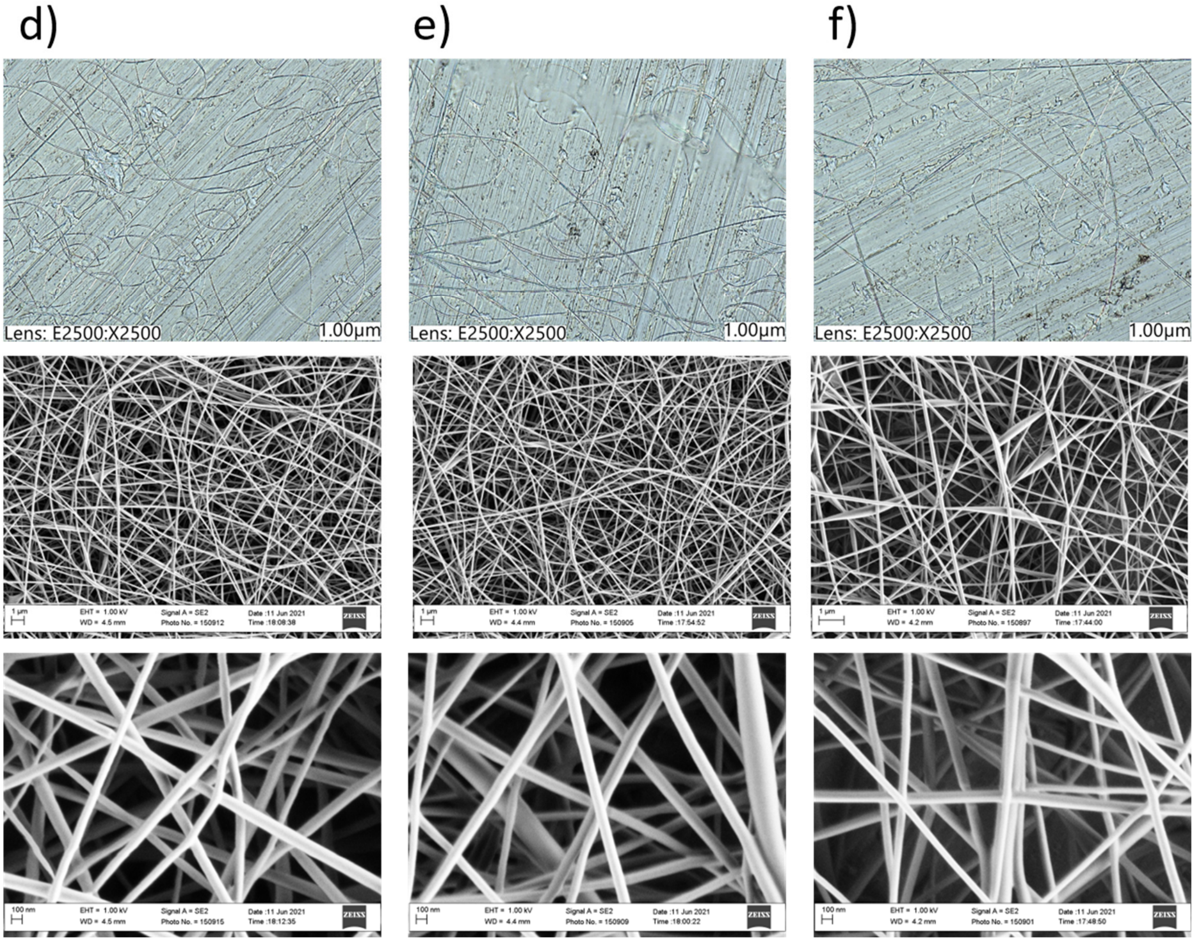
d)
e)
f)
Lens: E2500:X2500
1.00µm
Lens: E2500:X2500
1.00µm
Lens: E2500:X2500
1.00µm
1 µm
EHT = 1.00 kV
WD = 4.5 mm
Signal A = SE2
Photo No. = 150912
Date :11 Jun 2021
Time :18:08:38
1 µm
EHT = 1.00 kV
WD = 4.4 mm
Signal A = SE2
Photo No. = 150905
Date :11 Jun 2021
Time :17:54:52
1 µm
EHT = 1.00 kV
WD = 4.2 mm
Signal A = SE2
Photo No. = 150897
Date :11 Jun 2021
Time :17:44:00
100 nm
EHT = 1.00 kV
WD = 4.5 mm
Signal A = SE2
Photo No. = 150915
Date :11 Jun 2021
Time :18:12:35
100 nm
EHT = 1.00 kV
WD = 4.4 mm
Signal A = SE2
Photo No. = 150909
Date :11 Jun 2021
Time :18:00:22
100 nm
EHT = 1.00 kV
WD = 4.2 mm
Signal A = SE2
Photo No. = 150901
Date :11 Jun 2021
Time :17:48:50

g) h)

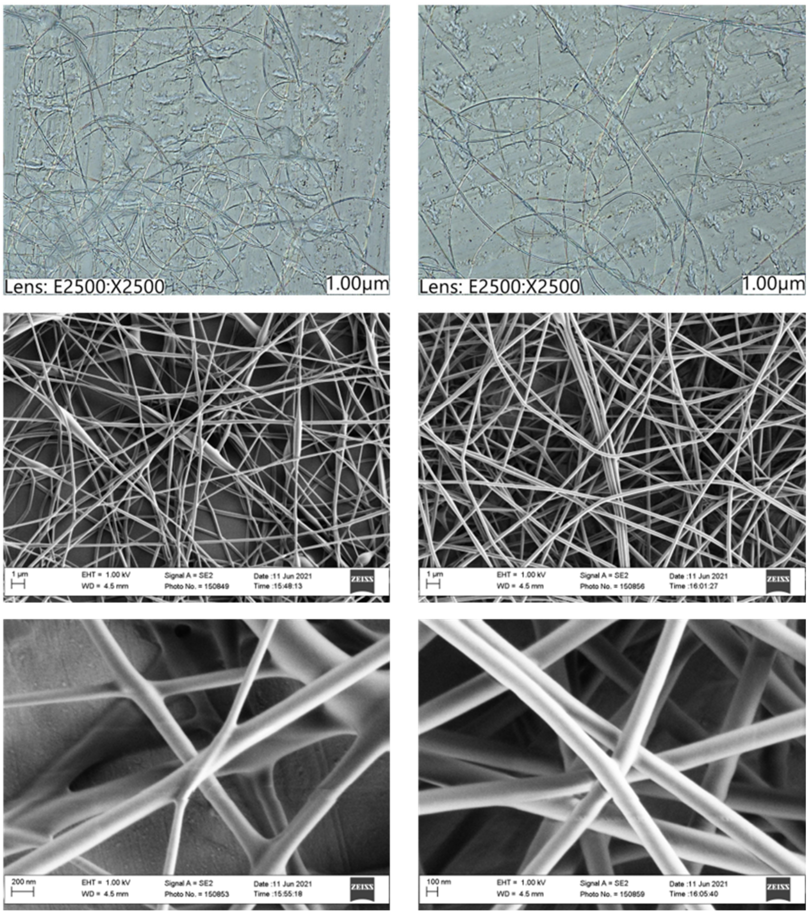
Lens: E2500:X2500
1.00µm
Lens: E2500:X2500
1.00µm
1 µm
EHT = 1.00 kV
WD = 4.5 mm
Signal A = SE2
Photo No. = 150849
Date :11 Jun 2021
Time :15:48:13
1 µm
EHT = 1.00 kV
WD = 4.5 mm
Signal A = SE2
Photo No. = 150856
Date :11 Jun 2021
Time :16:01:27
200 nm
EHT = 1.00 kV
WD = 4.5 mm
Signal A = SE2
Photo No. = 150853
Date :11 Jun 2021
Time :15:55:18
100 nm
EHT = 1.00 kV
WD = 4.5 mm
Signal A = SE2
Photo No. = 150859
Date :11 Jun 2021
Time :16:05:40

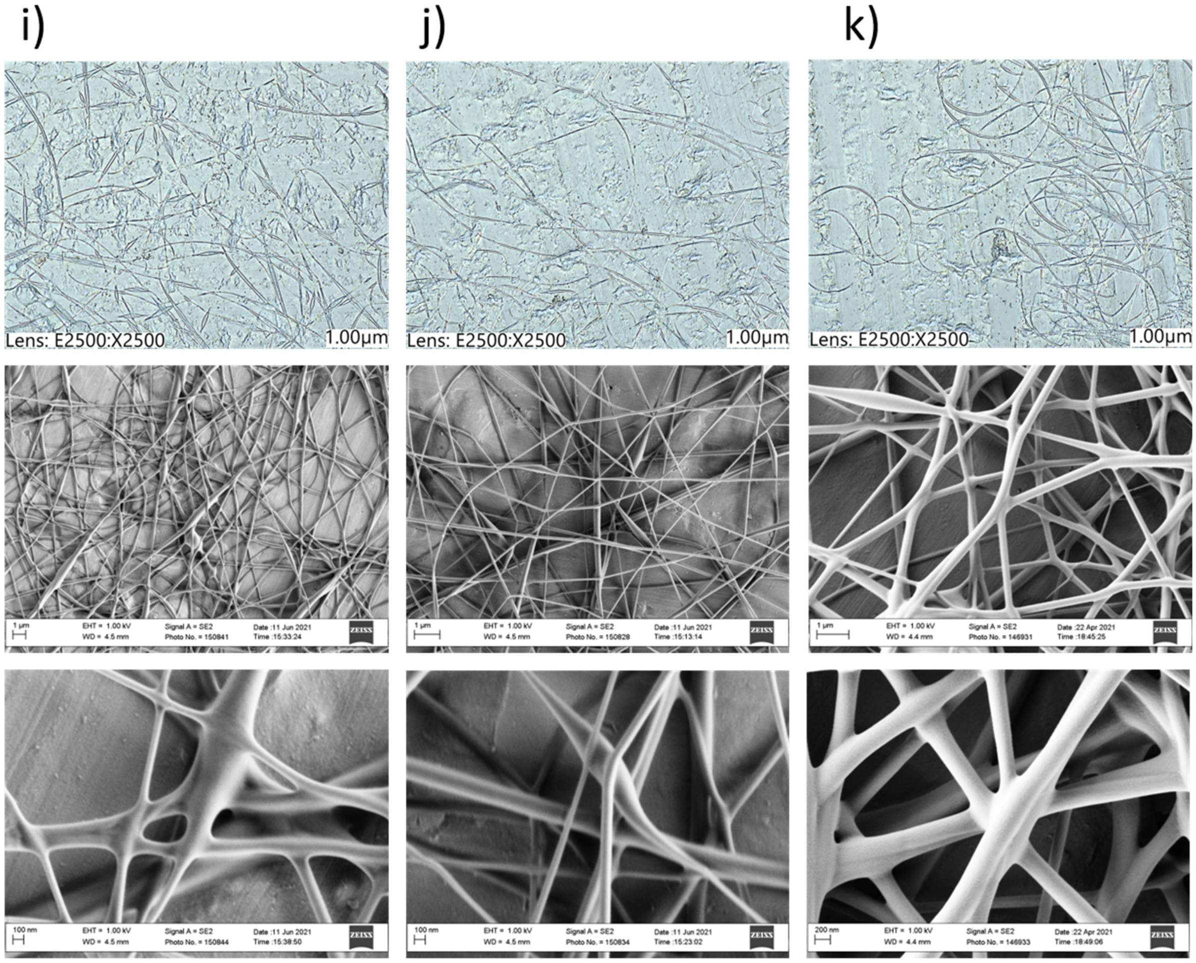
i)
j)
k)
Lens: E2500:X2500
1.00µm
Lens: E2500:X2500
1.00µm
Lens: E2500:X2500
1.00µm

l) m) n)

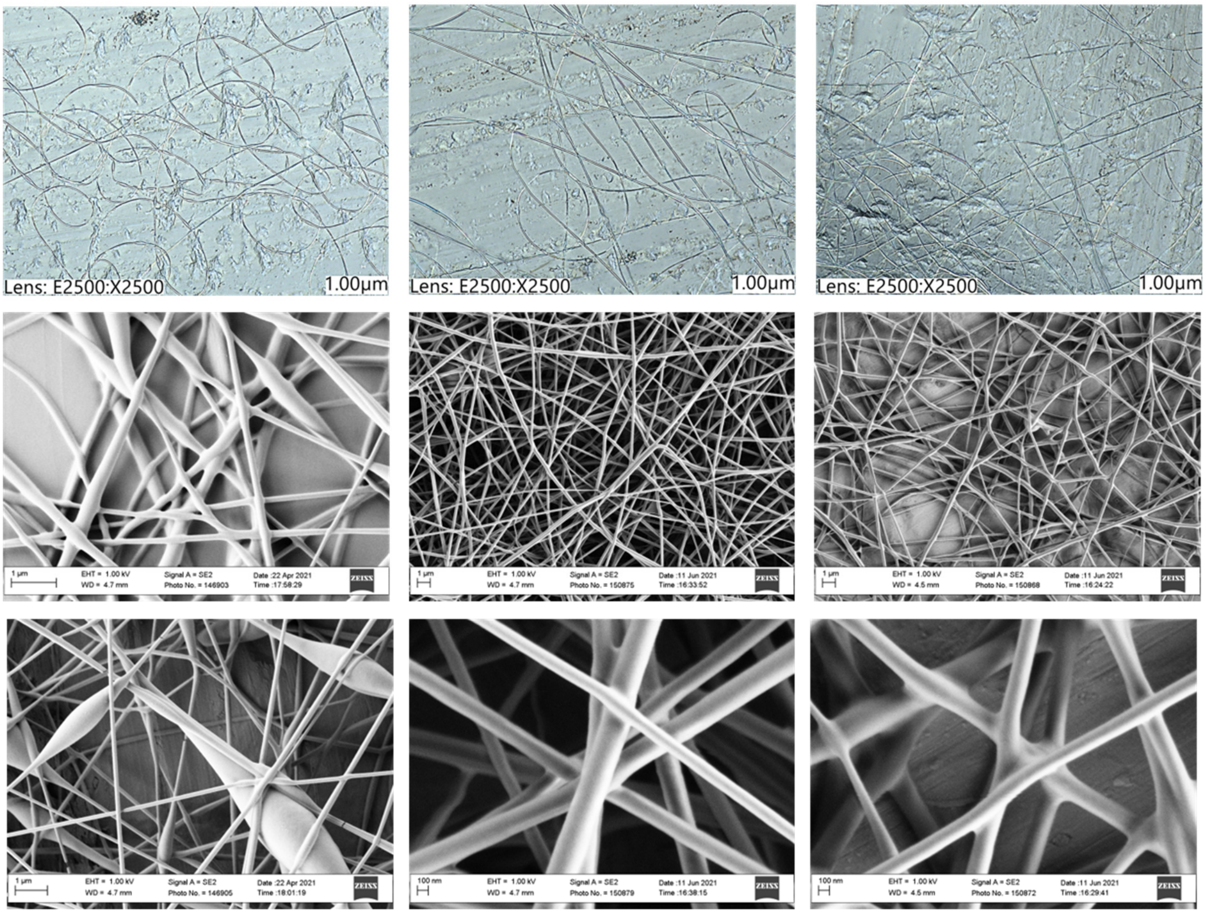
Lens: E2500:X2500
1.00µm
Lens: E2500:X2500
1.00µm
Lens: E2500:X2500
1.00µm
1 µm
EHT = 1.00 kV
WD = 4.7 mm
Signal A = SE2
Photo No. = 146903
Date :22 Apr 2021
Time :17:58:29
ZEISS
1 µm
EHT = 1.00 kV
WD = 4.7 mm
Signal A = SE2
Photo No. = 150875
Date :11 Jun 2021
Time :16:33:52
ZEISS
1 µm
EHT = 1.00 kV
WD = 4.5 mm
Signal A = SE2
Photo No. = 150868
Date :11 Jun 2021
Time :16:24:22
ZEISS
1 µm
EHT = 1.00 kV
WD = 4.7 mm
Signal A = SE2
Photo No. = 146905
Date :22 Apr 2021
Time :18:01:19
ZEISS
100 nm
EHT = 1.00 kV
WD = 4.7 mm
Signal A = SE2
Photo No. = 150879
Date :11 Jun 2021
Time :16:38:15
ZEISS
100 nm
EHT = 1.00 kV
WD = 4.5 mm
Signal A = SE2
Photo No. = 150872
Date :11 Jun 2021
Time :16:29:41
ZEISS

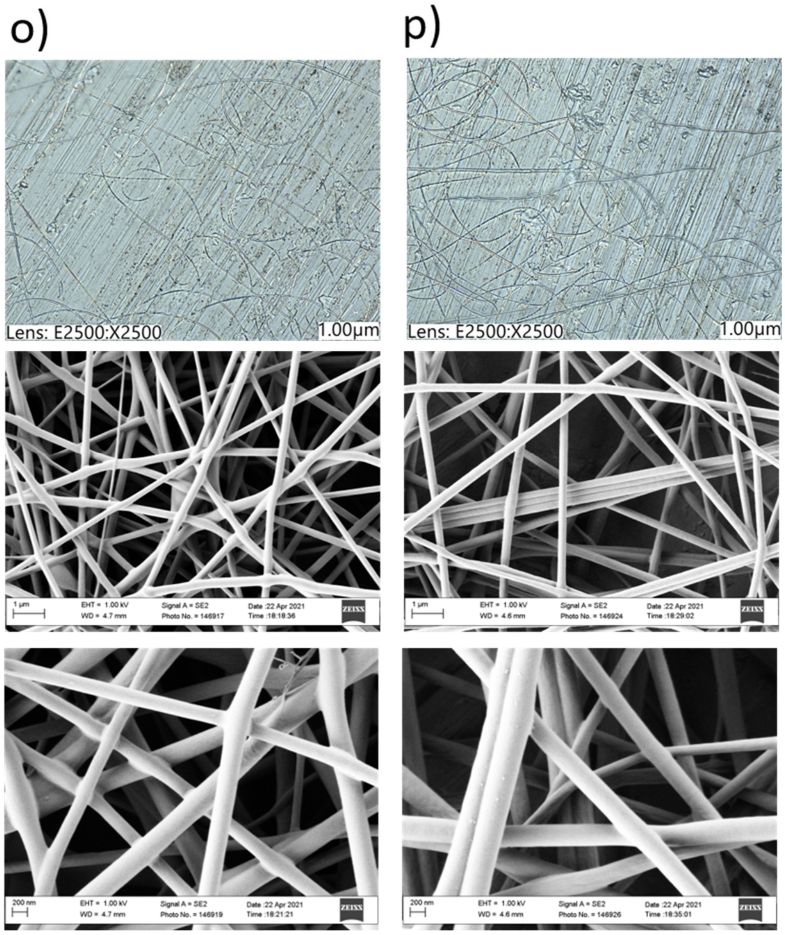

Figure S4: Fiber morphology: *Optical microscope (upper row) and SEM images (lower rows) of a) PAA_10, b) PAA_15, c) PAA_ 20, d) PAA-0.5SA_10, e) PAA-0.5SA_15, f) PAA-0.5SA_20, g) PAA-1SA_15, h) PAA-1SA_20, i) PAA-2.5SA_10, j) PAA-2.5SA_15, k) PAA-2.5SA_20, l) PAA-5SA_10, m) PAA-5SA_15, n) PAA-5SA_20, o) PAA-10SA_15, p) PAA-10SA_20*

References

1. Liu, H. *et al.* Effect of annealing and pressure on microstructure of cornstarches with different amylose/amylopectin ratios. *Carbohydr. Res.* **344**, 350–354 (2009).

2. Wu, X., Liu, P., Ren, L., Tong, J. & Zhou, J. Optimization of corn starch succinylation using response surface methodology. *Starch/Staerke* **66**, 508–514 (2014).

3. Zhao, H., Wu, X., Tian, W. & Ren, S. Synthesis and thermal property of poly(allylamine hydrochloride). *Adv. Mater. Res.* **150–151**, 1480–1483 (2011).

4. Kyzas, G. Z., Siafaka, P. I., Bikiaris, D. N., Koukaras, E. N. & Froudakis, G. E. Alternative use of cross-linked polyallylamine (known as Sevelamer pharmaceutical compound) as biosorbent. *J. Colloid Interface Sci.* **442**, 49–59 (2015).
